# Innovative Management of Opioid-Induced Hyperalgesia Using Lidocaine Infusion: A Case Report

**DOI:** 10.7759/cureus.66376

**Published:** 2024-08-07

**Authors:** Andrei Bautista, Ryan Ferguson, Praveen Reddy Elmati, Alexander Bautista

**Affiliations:** 1 Anesthesiology, duPont Manual High School, Louisville, USA; 2 Anesthesiology, University of Louisville, Louisville, USA; 3 Anesthesiology, Saint Clare’s Health, Denville, USA; 4 Anesthesiology, University of Louisville Hospital, Louisville, USA

**Keywords:** pain management, opioid management, neuropathic pain, lidocaine infusion, opioid-induced hyperalgesia

## Abstract

Prolonged opioid use carries risks, including addiction and dependence. A significant consequence of chronic opioid use is opioid-induced hyperalgesia (OIH), where patients experience heightened pain sensitivity. Managing OIH typically involves reducing opioid intake while mitigating withdrawal symptoms. This case report presents a patient with OIH treated with intravenous lidocaine and morphine. OIH presents complex pain management challenges, and lidocaine infusion has shown promise in mitigating its effects. Further research is needed to comprehensively assess the efficacy and safety of this treatment approach for patients with OIH.

## Introduction

Opioids are widely used to treat pain, with over 60 million users in 2021 [1]. However, prolonged use can lead to addiction, dependence, and paradoxically, increased pain sensitivity - a condition known as opioid-induced hyperalgesia (OIH) [2]. Treatment strategies for OIH involve reducing opioid intake while providing opioid antagonists to manage withdrawal symptoms [3]. Lidocaine infusion, used since 1943, has been increasingly applied in chronic pain management due to its mechanisms, including sodium channel modulation and reducing nociceptor sensitization [4]. This report presents a patient with OIH treated with a lidocaine infusion and morphine. We also discuss current literature on the use of systemic lidocaine in different types of pain. Informed consent was obtained before publication of this case report. 

## Case presentation

A 31-year-old female with a history of cardiac arrest and subsequent stroke and ventilator dependence through tracheostomy tube presented with Stevens-Johnson Syndrome-Toxic Epidermal Necrolysis Overlap Syndrome (SJS-TEN), which developed as a secondary reaction to antibiotic administration. She experienced severe, widespread pain due to extensive skin lesions and was initially treated with continuous fentanyl infusions with the dose gradually increasing to 200 mcg/hour. Additionally, she was started on scheduled intravenous (IV) hydromorphone at 3 mg every three hours, with an additional 3 mg of IV hydromorphone as needed for breakthrough pain. Despite these high doses of opioid medications, her pain was not adequately controlled, leading to a consultation with the inpatient pain medicine team. It is important to note here a trial of ketamine boluses was also attempted; however, due to a shortage of this medication, it was discontinued, and its efficacy could not be assessed.

To better assess her pain, the Critical-Care Pain Observation Tool (CPOT) was reviewed, revealing an average CPOT score of 7 at the time of consultation. Notably, despite the increased opioid dosing, her pain was worsening when compared to prior CPOT scores of 4-5, suggesting the possibility of OIH. This condition, characterized by increased sensitivity to pain despite escalating opioid dosages, was considered in her treatment plan.

Based on our recommendations, the fentanyl drip was discontinued, and she was started on IV morphine at 1.5 mg/hour, along with a concurrent lidocaine infusion at 1.5 mg/kg/hour. Daily lidocaine levels were monitored to achieve a therapeutic range of 4-5 µg/mL. Following this new treatment regimen, the patient experienced improved sleep quality and appeared more comfortable during examinations. Within 72 hours of initiating the lidocaine infusion, her CPOT score decreased significantly from an average of 7 to 3. As her lidocaine levels reached the therapeutic range, the morphine infusion rate gradually decreased while maintaining effective pain control. The lidocaine was discontinued after three days. No adverse effects were reported with this treatment protocol.

## Discussion

OIH diagnosis was made after ruling out all other causes for pain unrelieved by opioids, including worsening pain causes, opioid tolerance, withdrawal from opioids, and pseudo-addiction. OIH is a paradoxical response where prolonged opioid use leads to increased pain sensitivity, complicating pain management [2]. Diagnosing OIH involves distinguishing it from opioid tolerance and other pain conditions. Key diagnostic steps include clinical history and pain assessment, differentiating OIH from tolerance, exclusion of other causes, and provocative testing [5]. Assessing opioid use history, dosage, and duration is one of the first steps to identifying OIH. Increased pain sensitivity despite escalating opioid doses suggests OIH. Tools like the Numerical Rating Scale (NRS) and the CPOT can quantify pain levels. In this case, the patient exhibited increased pain despite higher opioid doses, with CPOT scores rising from 4-5 to 7. Unlike tolerance, which requires higher doses for the same effect, OIH is characterized by increased pain sensitivity despite escalating opioid doses. This was evidenced by the patient’s increasing CPOT scores despite the increase of opioids in the pain regimen. Other pain causes can be ruled out through imaging, lab tests, and clinical examination. Improvement of pain by reducing opioid doses can support an OIH diagnosis. In this presentation, reducing the fentanyl infusion and initiating a lidocaine infusion provided significant pain relief, supporting the OIH diagnosis.

OIH mechanisms include central sensitization, peripheral sensitization, neuroinflammation, and altered pain modulation. Chronic opioid use leads to changes in the central nervous system (CNS), resulting in hyperalgesia and allodynia. This involves N-methyl-D-aspartate (NMDA) receptor activation and increased excitatory neurotransmitter release. Opioids can also sensitize peripheral nociceptors, increasing pain perception through the upregulation of pain receptors and inflammatory mediators [5].

Managing OIH includes several strategies, such as opioid rotation, opioid dose reduction, NMDA antagonists, adjuvant analgesics, and lidocaine infusion [5]. Switching to different opioids with varying receptor affinities and pharmacokinetic profiles can alleviate OIH. Gradual tapering helps reduce OIH symptoms while avoiding withdrawal. This was evidenced by the successful reduction of the patient’s opioid dosage in this case. Medications like ketamine and methadone are NMDA receptor antagonists that inhibit central sensitization and reduce hyperalgesia. Although ketamine was initially administered, it was discontinued due to shortages in this patient. Non-opioid medications such as gabapentin, pregabalin, duloxetine, and amitriptyline manage pain and reduce opioid requirements. IV lidocaine modulates sodium channels, reduces peripheral nociceptor sensitization, and relieves central pain. It is cost-effective, easy to administer, and has a strong safety profile when monitored.

Lidocaine is an amide-based local anesthetic that works on peripheral sensitization by blocking voltage-gated sodium channels at low concentrations thus preventing aberrant expression and activation of sodium channels of damaged neurons. Some literature states it works by NMDA antagonism and modulates central sensitization to produce anti-hyperalgesic effects. Lidocaine also has anti-inflammatory properties and can reduce inflammatory cytokines which are postulated to cause secondary hyperalgesia and central sensitization [4,6].

Anti-hyperalgesic effects are only noted with IV infusions and in a dose-dependent manner. Few studies and case reports show evidence of systemic lidocaine reducing opioid consumption and providing pain relief. There is a lack of high-quality evidence of the benefit of lidocaine in OIH. Systemic lidocaine has been studied to relieve OIH in sickle cell disease during vaso-occlusive crisis, cancer pain, complex regional pain syndrome, chronic neuropathic pain, and perioperative pain.

In a study by Latika et al., a loading dose of 2 mg/kg of 8 mg/mL lidocaine was given over 30 minutes followed by a continuous infusion of 1-2 mg/kg/hour. This resulted in reduced use of opioids compared to control in two patients [7]. Nguyen et al. reported 15 patients with a trial of lidocaine infusion for patients with sickle cell disease [8]. The authors gave a challenge of 100 mg of lidocaine over 30 minutes followed by an IV infusion of lidocaine at 0.5mg/kg/hour until a maximum of 2 mg/kg/hour. Eight patients reported a reduction in pain scores by 20% or more. There was a 32% reduction in the morphine drug equivalent after lidocaine infusion. Lidocaine was continued for two to eight days in these patients. One patient also had three successful trials of IV lidocaine, enabling discontinuation of IV opioids and transition to oral opioid medications. Some authors reported the addition of ketamine along with lidocaine for pain relief.

Kajiume et al. described a combination of lidocaine, ketamine, and fentanyl use in a pediatric neuropathic cancer patient for pain relief that was allergic to morphine [9]. The authors used a dosage of 9 -14 μg/kg/minute, which was much lower than 25 to 83 μg/kg/minute used in other studies mentioned earlier. However, the authors reported efficacy and better pain control due to its combination with ketamine. Zanza et al. described 14 patients with severe postoperative pain who failed to respond to opioid pain medications [10]. Magnesium lidocaine and ketorolac cocktail improved the visual-analog pain score (VAS) and decreased the MME doses/hour in the first 12 hours after administration of the postoperative period.

Massey et al. discussed a case report on lidocaine infusion for terminal pediatric cancer patients with intractable pain [11]. Due to increasing opioid requirements, the authors started the patient on methadone and an infusion of lidocaine at 35-63 μg/kg/minute. The patient remained off opioid therapy and with significantly improved pain on lidocaine infusion for >2 months.

In a meta-analysis by Tremont-Lukats et al., lidocaine infusion relieved pain from diabetic polyneuropathy, post-herpetic neuralgia, and post-amputation stump pain [12]. The authors also reported that the studies usually measured the effect of this drug for 24 hours due to the short half-life of lidocaine of 120 minutes and its effects disappearing after a few hours. It is unknown why some studies showed improvement with chronic use of lidocaine. Often the lidocaine was used in combination with other neuropathic medications that could have contributed to the pain improvement on chronic use. Kandil et al. performed a literature review on lidocaine in various clinical settings [4]. The authors reported benefits in cancer pain and questionable benefits in postoperative pain.

In a Cochrane review by Weibel et al. on 68 studies and 4,525 participants, the authors could not find a significant difference between IV lidocaine and placebo in improving postoperative pain after 24 hours and were uncertain of a significant difference in the first 24 hours due to studies having a low quality of evidence [13]. Despite lidocaine having anti-inflammatory properties, as mentioned earlier, the pain in the postoperative period is primarily inflammatory and evidence in the literature shows lidocaine is not beneficial for inflammatory pain and improves pain from hyperalgesia or neuropathic pain. Lavand’homme et al. reported no difference in a randomized controlled trial of 85 participants with postoperative pain following major digestive surgery with a low dose of 0.5 mg/kg/hour [14]. Similar results were seen by Ortiz et al. in 44 patients with postoperative laparoscopic cholecystectomy pain [15]. Wilson et al. in their review stated the questionable benefit of systemic lidocaine in postoperative OIH [5].

Future studies need to focus on the type of pain, effective dosage, standardized testing scores/scale to document improvement and duration of benefit of lidocaine.

Adverse effects of lidocaine infusion include transient impairment of cognition, nausea, vomiting, abdominal pain, diarrhea, dizziness, and peri-oral numbness. Studies also reported acute blood pressure changes, muscle twitching, seizures, arrhythmias, metallic taste in the mouth, and insomnia [16,17]. Since the medication is dealkylated by the CYP3A4 in the liver, there is a risk of toxicity in patients with impaired hepatic function [18]. Blockage of sodium channels by lidocaine may be responsible for cardiac arrhythmias, bradycardia, QRS prolongation, and vascular collapse [19].

## Conclusions

This case report underscores the presence of OIH following high-dose opioid use and significant symptom improvement with lidocaine infusion. Lidocaine infusion is straightforward and cost-effective and has a strong safety record with mitigatable side effects. It can be particularly advantageous in resource-constrained environments. While the exact mechanisms are not fully understood, lidocaine's diverse actions make it a promising treatment for OIH. While lidocaine infusion shows promise, further research is needed to evaluate its effectiveness and safety comprehensively for patients with OIH.
